# Does Cognitive Behavior Therapy for psychosis (CBTp) show a sustainable effect on delusions? A meta-analysis

**DOI:** 10.3389/fpsyg.2015.01450

**Published:** 2015-10-06

**Authors:** Stephanie Mehl, Dirk Werner, Tania M. Lincoln

**Affiliations:** ^1^Department of Psychiatry and Psychotherapy, Philipps-University Marburg Marburg, Germany; ^2^Department of Health and Social Work, Frankfurt University of Applied Science Frankfurt, Germany; ^3^Department of Psychological Methods and Statistics, University of Hamburg Hamburg, Germany; ^4^Department of Clinical Psychology and Psychotherapy, University of Hamburg Hamburg, Germany

**Keywords:** CBT, CBTp, delusions, paranoia, follow-up

## Abstract

Cognitive Behavior Therapy for psychosis (CBTp) is an effective treatment resulting in small to medium effect sizes with regard to changes in positive symptoms and psychopathology. As a consequence, CBTp is recommended by national guidelines for all patients with schizophrenia. However, although CBTp was originally developed as a means to improve delusions, meta-analyses have generally integrated effects for positive symptoms rather than for delusions. Thus, it is still an open question whether CBTp is more effective with regard to change in delusions compared to treatment as usual (TAU) and to other interventions, and whether this effect remains stable over a follow-up period. Moreover, it would be interesting to explore whether newer studies that focus on specific factors involved in the formation and maintenance of delusions (causal-interventionist approach) are more effective than the first generation of CBTp studies. A systematic search of the trial literature identified 19 RCTs that compared CBTp with TAU and/or other interventions and reported delusions as an outcome measure. Meta-analytic integration resulted in a significant small to medium effect size for CBTp in comparison to TAU at end-of-therapy (*k* = 13; $\bar{d}$ = 0.27). However, the comparison between CBTp and TAU after an average follow-up period of 47 weeks was not statistically significant (*k* = 12, $\bar{d}$ = 0.16). When compared with other interventions, there was no significant effect of CBTp at end-of-therapy (*k* = 8; $\bar{d}$ = 0.16) and after a follow-up period (*k* = 5; $\bar{d}$ = −0.04). Comparison between newer studies taking a causal-interventionist approach (*k* = 4) and first-generation studies showed a difference of 0.33 in mean effect sizes in favor of newer studies at end-of-therapy. The findings suggest that CBTp is superior to TAU post-therapy in bringing about a change in delusions, but that this change may not be maintained over the follow-up period. Moreover, interventions that focus on causal factors of delusions seem to be a promising approach to improving interventions for delusions.

## Introduction

Before Cognitive Behavior Therapy for psychosis (CBTp) was introduced in the early 1990s, there was much concern that targeting delusions directly was likely to make matters worse. At the root of this concern was the assumption that psychotic symptoms such as delusions are qualitatively different from normal experiences and are therefore not amenable to reason or normal mechanisms of learning (Jaspers, 1913). Meanwhile, this view has been questioned by epidemiological studies that point to a continuum between normal and psychotic experiences (McGovern and Turkington, 2001; van Os et al., 2009) which indicates that normal reasoning could be involved in the formation and maintenance of delusional beliefs. This view, along with research on cognitive and emotional correlates of psychotic symptoms (Garety et al., 2001) has been one of the main suppositions upon which the systematic development of CBTp is based. CBTp was adapted from cognitive therapy, which was originally developed by A. T. Beck to treat depression (Beck, 2005). A characteristic aspect of CBTp compared to other psychological interventions for psychosis (e.g., psychoeducation, skill trainings etc.) is that the therapist works directly with delusional beliefs, not only by challenging the beliefs suspected of triggering and maintaining them (e.g., beliefs about the self and others) but also by questioning the delusional beliefs *per se*.

In the last 20 years, about 50 randomized controlled therapy studies (identified in a recent short review: Naeem et al., 2014) have demonstrated that CBTp is an effective adjunct to standard care. CBTp generally reduces positive symptoms, negative symptoms, general functioning and symptoms of depression (Gould et al., 2001; Rector and Beck, 2001; Zimmermann et al., 2005; Wykes et al., 2008; Sarin et al., 2011). Several national guidelines thus recommend CBTp for patients with schizophrenia in all phases of the disorder (DGPPN, 2006; NICE, 2014).

Despite the plentiful research on CBTp, the degree to which CBTp affects delusions as such has remained unclear. This is because the intervention studies generally used broader outcome measures of positive symptoms or general psychopathology as the primary outcome measure rather than delusions. Somewhat surprisingly, it was not until recently that researchers first attempted to address the question of how effective CBTp is in changing delusions as such. Van der Gaag et al. (2014) did this by analysing effects from secondary outcome measures of RCTs on CBTp. They included nine RCTs (from a total of 50 RCTs of CBTp) that had reported on change in delusions and found a significant, but small to medium effect of CBTp on delusions *(*$\bar{d}=$ 0.36, 95%-CI: 0.08, 0.63). However, due to the fairly narrow definition of individually tailored formulation-based CBTp, several RCTs evaluating CBTp were excluded (Cather et al., 2005; Turkington et al., 2006; Garety et al., 2008; Foster et al., 2010). Moreover, follow-up data were not analyzed. Thus, it would be interesting to see whether the effect remains significant if broader inclusion criteria are used. Also, it remains open whether change in delusions is sustainable over a follow-up period.

Finally, van der Gaag et al. (2014) excluded some of the more recent studies (Foster et al., 2010) that used a quite interesting approach with regard to change in delusions: an interventionist-causal model approach (Kendler and Campbell, 2009). This approach selects one of several cognitive and emotional factors that are hypothesized to be involved in the formation and maintenance of delusions (Freeman, 2007; Garety et al., 2007; Freeman and Garety, 2014) and aims to change this factor by means of cognitive-behavioral interventions that target this factor but do not challenge the delusion itself. For example, Freeman and colleagues targeted worrying by employing several interventions: (1) psychoeducation on worry, (2) identification and reviewing of positive and negative beliefs about worry, (3) increasing awareness of individual triggers of worry, (4) planning activity at times of worry, and learning to let go of worry (Freeman et al., 2015).

Thus, this meta-analysis tests whether CBTp has any benefits in comparison to (1) standard care and (2) other psychological treatments such as supportive therapy, problem solving, and family interventions and (3) whether its effects are still present after a follow-up period. (4) Finally, it explores whether newer cognitive-behavioral interventions that take a causal-interventionist approach by focusing solely on specific factors involved in the formation and maintenance of delusions are more effective in changing delusions than the first generation of CBTp studies.

## Methods

### Eligibility criteria

To be included, studies had to be: (1) randomized controlled trials assessing (2) individualized CBTp for psychosis compared to (3) treatment as usual (TAU) or other psychological interventions (such as family interventions, supportive therapy, problem solving) in (4) patients with a psychotic disorder (at least 75% of the sample), be (5) published in peer-reviewed journals and report (6) on change in delusions using a reliable scale. (7) We excluded studies focusing on a specific subgroup of patients such as those with a comorbid substance disorder. CBTp was defined according to the criteria of the National Institute of Health and Clinical Excellence (NICE, 2014): (1) links are established between patients thoughts, feelings or actions and their current or past symptoms and functioning, (2) patient perceptions, beliefs or reasoning are reevaluated in relation to target symptoms. TAU or standard care included regular outpatient appointments with psychiatrists and prescription of medication. In contrast, supportive therapy included weekly sessions with a therapist who used basic therapeutic skills such as listening, reflecting, empathizing, and summarizing.

### Information sources and search

Relevant studies were identified by an electronic literature search using five databases: MEDLINE, EMBASE, Cochrane Central Register of Controlled Trials, PsycINFO, and PsycLIT from 1987 to 21st January 2015 in the English or German languages. Published meta-analyses and reviews were also searched.

**Figure 1:**
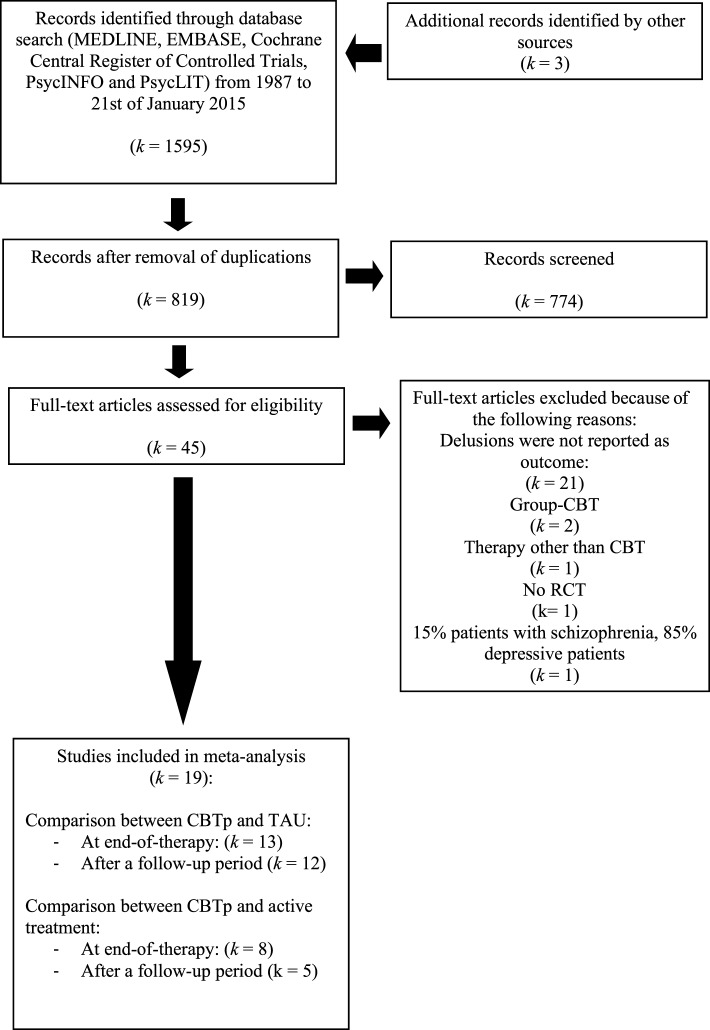
**Flow chart of selected studies**.

We conducted three different searches that were combined later. First, we searched the databases on the terms “CBT” OR “cognitive therapy” OR “cognitive behavioural therapy” OR “cognitive behavior therapy” OR “cognitive behaviour therapy” OR “cognitive behavior therapy.” Second, we searched the databases on “psychosis” OR “psychotic symptoms” OR “schizophreni^*^” OR “paranoi^*^.” Third, we investigated the terms “RCT” OR “randomized controlled trial” OR “randomised controlled trial.” Then, we combined all three searches, using the operator AND, which yielded 1598 studies. Removing duplicates resulted in 816 studies (see flow chart depicted on Figure 1). Of these, 774 could be excluded beyond doubt after reading the title, leaving 42 studies. The search of existing meta-analyses identified three further studies. The remaining 45 studies were read by the first author and a Master's student of clinical psychology. Of these, 19 studies fulfilled our inclusion criteria and were ultimately included.

### Statistical analysis

Study characteristics and the appropriate statistics to calculate effect sizes were independently coded by the first and second authors. Statistical analyses were carried out in *R* (Version 3.1.2) using the meta-analysis package metafor (Version 1.9-5). We calculated the bias-corrected standardized mean difference *(d)* on all delusion-related outcomes for every treatment-control group comparison using the pooled standard deviation as the standardizer (Hedges and Olkin, 1985). A positive sign for *d* indicates that the CBTp group was better off after treatment compared to the control condition. If a study reported results for subscales or for more than one delusion-related outcome, we calculated a single composite effect size for each study to be able to analyze stochastically independent effect size estimates. Effect sizes were calculated on the basis of pretest data, posttest data, and at follow-up if appropriate statistics were available. We used end-of-treatment statistics (controlled for the smaller number of patients at follow-up) for one study that reported that there were “no significant differences” between end-of-treatment and follow-up scores but did not report the scores (Pinninti et al., 2010). Whenever a study reported more than one follow-up measurement, we calculated the effect size for the final measurement in order to estimate the long-term effects of treatment.

We did not assume that all included studies share a common effect size, because the studies obviously differ in various ways (e.g., duration of treatment, format of therapy, experience of therapists, patient population). To allow for variation in true effect sizes (δ_*i*_) we fitted a random-effects model to the data and estimated the amount of heterogeneity with restricted maximum-likelihood estimation (Raudenbush, 2009).

We conducted two meta-analyses: one of all available comparisons of CBTp vs. TAU and one of all available comparisons of CBTp vs. other psychological interventions. For each analysis we report the estimated mean population effect size (${\hat{\mu }}_{\delta }$ subsequently denoted as $\bar{d}$), the *p*-value for the test H0: μδ = 0, the estimated variance of the true effect sizes (${\hat{\tau }}^{2}$), the results for the Q-test for heterogeneity with a *p*-value for the test H0: τ^2^ = 0. As the number of included studies might be quite small and the Q-test might have low statistical power in order to test for heterogeneity, we also reported an I^2^-statistic to estimate the percentage of observed variation in effect sizes that is due to hetereogeneity, as recommended by Deeks et al. (2008). In order to compare newer studies that used a causal-interventionist approach with first-generation CBTp studies, we performed a subgroup analysis and calculated the mean effect size at end-of-treatment for (a) the studies that used the causal-interventionist approach and (b) for all other studies. Then we calculated the difference between both mean effect sizes. In addition, 95% confidence intervals were calculated for all above-mentioned statistics.

We investigated the possibility of publication bias with funnel-plots and regression-tests (Sterne and Egger, 2005). We used a trim-and-fill analysis (Duval, 2005) to investigate the impact of missing studies on the overall results.

## Results

### Descriptive information on included studies

Fourteen studies were identified that compared CBTp with TAU (see flow-chart on Figure 1 and Table 1 for more information on the studies) and eight studies that compared CBTp with other psychological interventions. Three studies (Lewis et al., 2002; Durham et al., 2003; Garety et al., 2008) reported results for one CBTp and two control conditions and were included in both meta-analyses.

**Table 1:** Studies included in the comparison of CBTp vs. TAU and CBTp vs. other psychological interventions: description of the intervention, patient characteristics and outcome measure.

**Author and Year**	**Subject characteristics: Experimental Condition (EC), Control Condition I (C1) Control Condition II (CCII)**	**Experimental condition (EC) CBT format patients**	**Control condition I (CC I) format patients**	**Control condition II (CC II)**	**Duration of intervention EC/CCI/CC II**	**Total no. of sessions, Mean number of sessions, EC/CC I/CC II**	**Selected outcome measure**	**Blind assessment?**	**ITT-data?**	**Follow-up**
Cather et al., 2005	Number of randomized patients: *n* = 28, Diagnoses: 17 SZ; 11 SA, Age: EC: *M* = 45.8 (*SD* = 10.2) CCI: *M* = 33.1 (*SD* = 10.3), Medication: EC: 100%/CCI: 100%	Functional CBT, Based on established manuals (Kingdon and Turkington, 1994; Fowler et al., 1995; Chadwick et al., 1996; Nelson, 1997), Number of randomized patients: *n* = 15	Psychoeducation, Number of randomized patients: (*n* = 15)		16/16 weeks	Total number of sessions: 16/16^6^	PSYRATS del.	Yes	No	–
Durham et al., 2003	Number of randomized patients *n* = 66, Diagnoses: 59 SZ; 5 SA; 2 DD, Age: EC: *M* = 36 (*SD* = 10.0)/CCI: *M* = 36 (*SD* = 10.2)/CC II: *M* = 37 (*SD* = 11.2), Medication: EC: 100%/CC I: 86%	CBT, Best practice based on established manuals (Tarrier, 1992; Kingdon and Turkington, 1994), Number of randomized patients: *n* = 22	TAU, Number of randomized patients: *n* = 21	Supportive therapy, Number of randomized patients: *n* = 23	39 weeks/–/22 weeks	Total number of sessions: EC: 20/–/CC II: 20, Mean number of sessions: EC: 14.8,/–/CC II: 16.8, D_sessions_ = −2.0	PSYRATS del.	Yes	No	52 weeks
Foster et al., 2010	Number of randomized patients *n* = 24, Diagnoses: SZ, SA, and DD^1^, Age: EC: 40.0 (10.5)/CC I: 39.1 (9.2), Medication: EC: 92%/CCI: 83%	Worry-CBT, Fixed sessions based on a manual (Wells, 1997), Number of randomized patients: *n* = 12	TAU, Number of randomized patients: *n* = 12		4 weeks/–	Total number of sessions: 4/–	PSYRATS del.	No	No	9 weeks
Freeman et al., 2015	Number of randomized patients: *n* = 150, Diagnoses: 111 SZ; 11 SA; 10 DD; 18 POS, Age: EC: 40.9 (10.5)/CC I: 42.1 (13.1), Medication: 94%^3^	Worry-CBT, Based on self-help manual, (Freeman and Freeman, 2013), Number of randomized patients: *n* = 73	TAU, Number of randomized patients: *n* = 77	–	8 weeks./–	Total number of sessions: 6/–, Mean number of sessions: EC: 5.5	PSYRATS del.	Yes	No	24 weeks
Freeman et al., 2014	Number of randomized patients: *n* = 30, Diagnoses: 22 SZ; 6 SA; 1 DD; 1 POS, Age: EC: 41.9 (11.5)/CC I: 41.5 (13.1), Medication: EC: 100%/CC I: 100%	Brief CBT, Based on self-help manual (Freeman and Freeman, 2012), Number of randomized patients: *n* = 15	TAU, Number of randomized patients: *n* = 15		8 weeks/–	Total number of sessions: 6/–, Mean number of session: EC: 6.67/–	PSYRATS del.	Yes	No	12 weeks
Garety et al., 2008	Number of randomized patients: *n* = 328, Diagnoses: 258 SZ; 38 SA; 5 DD, Age: n.r., Medication: n.r.	CBT (carer + no-carer), Based on an established manual (Fowler et al., 1995), Number of randomized patients: *n* = 160	TAU, Number of randomized patients (carer + no-carer): *n* = 140	Family intervention, Number of randomized patients: *n* = 28	39 weeks	Total number of sessions: 20/–, Mean number of sessions: EC: 14.3/–/CC II: 13.9, D_sessions_ = 0.4	PSYRATS del., conviction and delusion distress	Yes	No	52 weeks
Haddock et al., 2009	Number of randomized patients: *n* = 77, Diagnoses: 69 SZ; 7 SA; 1 POS, Age: EC: 35.7 (12.5)/CC I: 33.9 (9.7), Medication: EC: 100%/CC I: 100%	CBT, Based on an established manual (Haddock et al., 2004), Number of randomized patients: *n* = 38	Social activity therapy, Number of randomized patients: *n* = 38		26 weeks	Total number of sessions: 25, Mean number of sessions: EC: 13.13/CC I: 14.9, D_sessions_ = −1.77	PSYRATS del.	Yes	No	24 weeks
Kråkvik et al., 2013	Number of randomized patients: *n* = 55, Diagnoses: 34 SZ/2 SA/9 DD, Age: EC: 37.5 (11.2)/ CC I: 35.3 (8.9), Medication: EC: 100%/CC I: 100%	CBT, Simplified version of an established manual (Chadwick et al., 1996), Number of randomized patients: *n* = 23	TAU^2^, Number of randomized patients: *n* = 22	–	26 weeks	Total number of sessions: 20	PSYRATS cognitive and emotional	No	Yes	52 weeks^2^
Lewis et al., 2002	Number of randomized patients: *n* = 309, Diagnoses: 123 SZ; 109 SFD; 39 SA; 25 DD; 13 POS, Age: EC: 29.1/CC I: 27.0/CC II: 27.2^4^, Medication: EC: 100%/CC I: 100%/CC II: 100%	CBT, Based on an established manual (Haddock et al., 1999b), Number of randomized patients: *n* = 101	TAU, Number of randomized patients: *n* = 102	Supportive counseling Number of randomized patients: *n* = 106	5 weeks	Total number of sessions: 20, Mean number of sessions:EC: 16.1/–/CC II: 15.7, D_sessions_ = −0.4	PSYRATS del.	Yes	No	67 weeks
Lincoln et al., 2012	Number of randomized patients: *n* = 80, Diagnoses: 58 SZ; 13 SA; 5 DD; 4 APD, Age: EC: 33.2 (10.4)/CC I: 33.1 (10.9), Medication: EC: 100%/CC I: 97%	CBTp, Based on an established German manual (Lincoln, 2006), Number of randomized patients: *n* = 40	TAU^2^, Number of randomized patients: *n* = 40	–	38 weeks	No fixed number of sessions. Mean number of sessions EC: 29/–	PDI distress, preoccupation, conviction	No	Yes	52 weeks^2^
Morrison et al., 2014	Number of randomized patients: *n* = 74, Diagnoses: SZ, SA, and DD^1^, Age: EC: 33.0 (13.1)/CC I: 29.7 (11.9), Medication: EC: 0%/CC I: 0%	CBTp, Based on established manuals (Morrison et al., 2004; Kingdon and Turkington, 2005), Number of randomized patients: *n* = 37	TAU, Number of randomized patients: *n* = 37	–	39 weeks	Total number of sessions: 26, Mean number of sessions: EC: 13.3/–	PSYRATS cognitive and emotional	Yes	No	19 weeks
O'Connor et al., 2007	Number of randomized patients: *n* = 24, Diagnoses: 24 DD, Age: EC: 40.0 (9.4)/CC I: 36.8 (13.5), Medication: EC: 100%/CC I: 100%	CBTp, Based on established manuals (Fowler et al., 1995; Chadwick et al., 1996), Number of randomized patients: *n* = 12	Attention placebo control, Number of randomized patients: *n* = 12	–	24 weeks	Total number of sessions: 24	MADS	Yes	No	–
Pinninti et al., 2010	Number of randomized patients: *n* = 33, Diagnoses: 11 SZ; 22 SA, Age: 40.0 (11.0)^3^, Medication: EC: 100%/CC I: 100%	CBTp, Not manualized, Number of randomized patients: *n* = 18	TAU, Number of randomized patients: *n* = 15	–	12 weeks	Total number of sessions: 12, Mean number of sessions EC: 11.9/–	PSYRATS del.	Yes	No	24 weeks
Rathod et al., 2013	Number of randomized patients *n* = 35, Diagnoses: SZ, SA, and DD^1^, Age: EC: 31.4 (12.3)/CC I: 35.6 (10.7), Medication: EC: 100%/CC I: 100%	Culturally adapted CBTp Based on a study protocol (Rathod et al., 2010), Number of randomized patients: *n* = 17	TAU, Number of randomized patients: *n* = 15	–	18 weeks	Total number of sessions: 16, Mean number of sessions: EC: 13.6/–	CPRS del.	Yes	Yes	26 weeks
Tarrier et al., 1993	Number of randomized patients: *n* = 27, Diagnoses: 307 SZ, Age: EC: 42.8 (12.3)/CC I: 42.8 (12.3), Medication: EC: 100%/CC I: 100%	Coping strategy enhancement, Based on an established manual (Tarrier, 1992), Number of randomized patients: *n* = 15	Problem solving, Number of randomized patients: *n* = 12	–	5 weeks	Total number of sessions: 10	PAS delusions	No	No	31 weeks
Tarrier et al., 2014	Number of randomized patients *n* = 49, Diagnoses: SZ, SA, DD, POS^1^, Age: EC: 32.6 (11.7)/CC I: 37.3 (14.2), Medication: EC: 100%/CC I: 100%	CBT for suicidal patients, Based on a manual (Tarrier et al., 2013), Number of randomized patients: *n* = 25	TAU, Number of randomized patients: *n* = 24	–	12 weeks	Total number of sessions: 24	PSYRATS del.	Yes	No	17 weeks
Turkington et al., 2006	Number of randomized patients: *n* = 422, Diagnoses: 422 SZ, Age: n. r., Medication: EC: 100%/CC I: 100%	CBTp, Based on established manuals (Kingdon and Turkington, 1994, 2005), Number of randomized patients: *n* = 281	TAU^2^, Number of randomized patients: *n* = 141	–	10.5 weeks	Total number of sessions: Mean number of sessions: EC: 6/–	PSYRATS del.	Yes	No	52 weeks
Valmaggia et al., 2005	Number of randomized patients: *n* = 62, Diagnoses: 62 SZ, Age: EC: 35.4 (10.5)/CC I: 35.5 (11.4), Medication: EC: 100%/CC I: 100%	CBTp, Based on an established manual (Kingdon and Turkington, 1994), Number of randomized patients: *n* = 36	Supportive counseling, Number of randomized patients: *n* = 26	–	22 weeks	Total number of sessions: 16	PSYRATS cognitive and emotional scale	Yes	Yes	48 weeks
Waller et al., 2015	Number of randomized patients: *n* = 31, Diagnoses: 27 SZ, 2 SA, 2 DD, Age: EC: 39.1 (10.5)/CC I: 43.0 (10.7), Medication: EC: 90%/CC I: 91%	Focused CBT, Sessions described in the study, Number of randomized patients: *n* = 20	TAU, Number of randomized patients: *n* = 11	–	5 weeks	Total number of sessions: 4	PSYRATS del.	No	Yes	8 weeks

*M, Mean; SD, Standard deviation; TAU, Treatment as Usual; EC, Experimental condition; CCI, Control condition I; CCII, Control condition II; SZ, Schizophrenia; SA, Schizoaffective Disorder; DD, Delusional disorder; APD, Acute psychotic disorder; POS, Psychosis not otherwise specified; SFD, Schizophreniform disorder;Medication, percentage of patients treated with antipsychotic medication. PSYRATSdel., PSYRATS delusions score; CPRS, Comprehensive Psychopathology Rating Scale; PAS, Psychiatric Assessment Scale;n.r., not reported;Dsessions, Mean number of CBT sessions—Mean number of other therapy sessions*;

1 *no information on diagnosis ratio*;

2 *study was not included in follow-up comparison between CBTp and TAU,as the study used a wait-list design and comparisons between CBTp and TAU are not possible at follow-up assessment*;

3 *variable was only reported for all patients*;

4 *SD was not reported*.

Most studies (*n* = 18) used observer-rated assessments of delusions such as the Psychotic Symptom Rating Scale (*k* = 17; PSYRATS: Haddock et al., 1999a) or the Maudsley Assessment of Delusions Scale (*k* = 1; MADS: Wessely et al., 1993). Four of these studies did not use single-blind assessment (Tarrier et al., 1993; Foster et al., 2010; Kråkvik et al., 2013; Waller et al., 2015) and one study (Lincoln et al., 2012) used a self-report measure (Peters et al. Delusions Inventory: Peters et al., 1999). Most studies (*k* = 12) selectively included patients with delusions (Tarrier et al., 1993; Lewis et al., 2002; Durham et al., 2003; Valmaggia et al., 2005; O'Connor et al., 2007; Haddock et al., 2009; Foster et al., 2010; Kråkvik et al., 2013; Freeman et al., 2014, 2015; Morrison et al., 2014; Waller et al., 2015), but only one of these studies predefined change in delusions as the primary outcome (Waller et al., 2015).

Of the 14 studies that compared CBTp and TAU, most studies (Tarrier et al., 1993; Lewis et al., 2002; Durham et al., 2003; Garety et al., 2008; Haddock et al., 2009; Pinninti et al., 2010; Lincoln et al., 2012; Kråkvik et al., 2013; Morrison et al., 2014) used traditional CBTp based on established manuals (Kingdon and Turkington, 1994; Fowler et al., 1995; Chadwick et al., 1996; Lincoln, 2006). One study used a brief and more “technical” version of CBTp administered by trained nurses (Turkington et al., 2006), another used a culturally-adapted version of CBTp in a population of migrants (Rathod et al., 2013). Two studies assessed the effectiveness of CBTp in other specific populations, namely patients who refused to take antipsychotic medication (Morrison et al., 2014) and patients who reported suicide attempts or current suicidal ideation (Tarrier et al., 2014). Four studies used an interventionist causal model approach and focused on cognitive or emotional factors involved in the formation and maintenance of persecutory delusions: negative self-evaluations (Freeman et al., 2014), worrying (Foster et al., 2010; Freeman et al., 2015), and reasoning biases (Waller et al., 2015).

Patients received between 4 and 29 therapy sessions, the mean number of sessions was 14.8 (*SD* = 8.3 sessions) and the duration of treatment varied between 4 and 39 weeks with a mean duration of 19.9 weeks (*SD* = 14.7 weeks). Most of the studies (*k* = 13) reported results at *end-of-therapy*. One study was only included in comparisons after a *follow-up* period, as findings at *end-of-therapy* were not reported (Turkington et al., 2006). Two studies used a wait-list group that later received CBTp (Lincoln et al., 2012; Kråkvik et al., 2013). As they did no longer use a controlled design at *follow-up*, their results were not included in *follow-up* analysis. In sum, 12 studies were included in the comparison between CBTp and TAU after an average *follow-up* period of 46.8 weeks (*SD* = 58.5 weeks).

All studies that were included in comparisons between CBTp and other psychological interventions (*k* = 8) used traditional CBTp (Tarrier et al., 1993; Lewis et al., 2002; Durham et al., 2003; Cather et al., 2005; Valmaggia et al., 2005; O'Connor et al., 2007; Garety et al., 2008; Haddock et al., 2009) based on established manuals (Kingdon and Turkington, 1994; Fowler et al., 1995; Chadwick et al., 1996; Nelson, 1997; Haddock et al., 2004; Morrison et al., 2004) (see Table 1 and Figure 1 for more information on the studies). One study assessed the effectiveness of CBTp in patients with a history of violence (Haddock et al., 2009). Four of the studies compared CBTp with a therapy placebo such as supportive counseling/therapy (Lewis et al., 2002; Durham et al., 2003; Valmaggia et al., 2005) or attention placebo (O'Connor et al., 2007). Other studies used psychoeducation (Cather et al., 2005), problem solving (Tarrier et al., 1993), family intervention (Garety et al., 2008) and social activity therapy (Haddock et al., 2009). Patients received between 10 and 25 sessions of treatment. Average number of sessions was 16.81 (*SD* = 5.5). The average duration of treatment was 22 weeks (*SD* = 13.2 weeks). Only five studies (Lewis et al., 2002; Durham et al., 2003; Valmaggia et al., 2005; Garety et al., 2008; Haddock et al., 2009) reported comparisons between CBTp and other psychological interventions after a follow-up period with the average follow-up period being 34.4 weeks (*SD* = 23.0 weeks).

### Comparisons of CBTp and treatment as usual (TAU)

**Figure 2:**
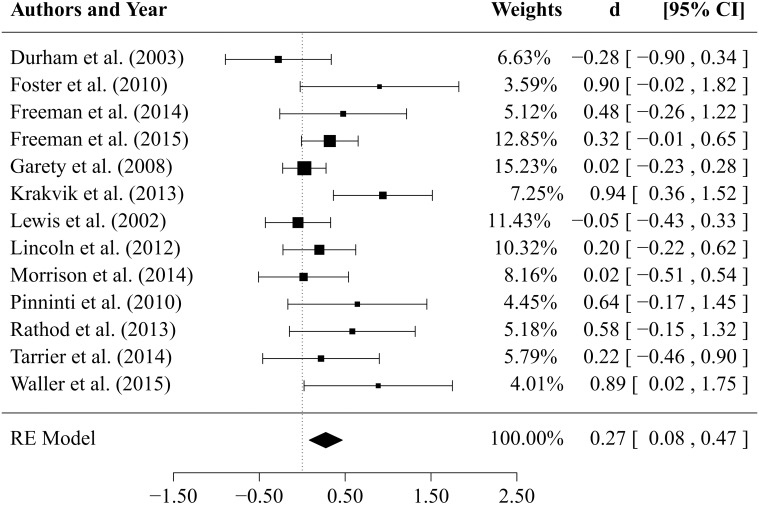
**Forest plot of effect sizes for the comparison between CBTp and treatment as usual (TAU) at end-of-therapy**.

Results of the comparisons between CBTp vs. TAU (*k* = 13 studies) at *end-of-therapy* are depicted in Figure 2 in form of a forest plot. The estimated mean effect size of CBTp was small to medium ($\bar{d}=$ 0.27, *SE* = 0.10, *p* = 0.005) with a 95% confidence interval ranging from 0.08 to 0.47. The estimator of the between-study variance revealed an estimate of ${\hat{\tau }}^{2}=$ 0.05 (95% CI: 0.00 to 0.32), the *Q*-statistic was non-significant (*Q* = 20.46, *df* = 12, *p* = 0.059). The small to medium value of *I*^2^ = 42.1% indicates that approximately 42% of the observed variance in effect sizes might be due to heterogeneity. However, one study (Kråkvik et al., 2013) had an especially large influence on the amount of observed heterogeneity. If we exclude this study, the proportion of observed variance due to real differences in effect sizes drops to approximately 12% (*I*^2^ = 11,7%).

**Figure 3:**
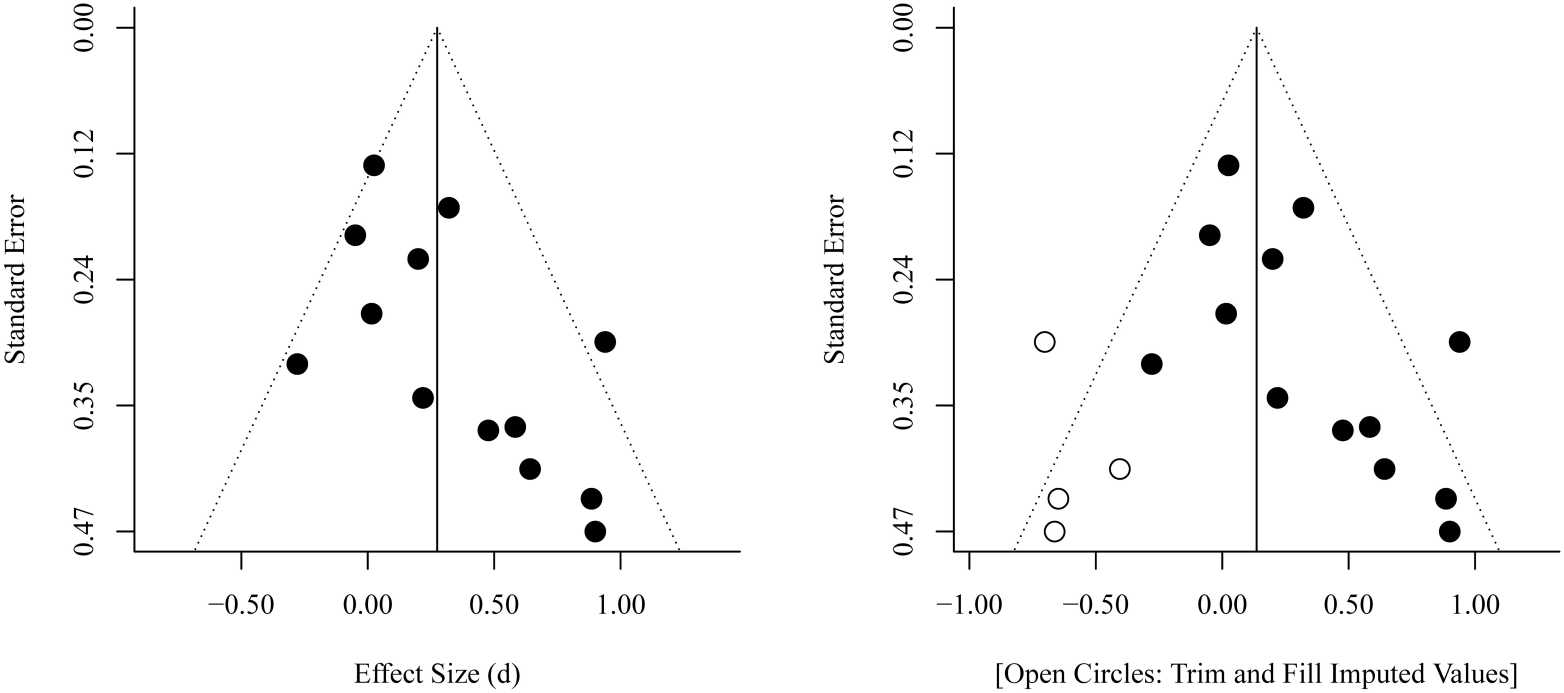
**Funnel Plots for the comparison between CBTp and treatment as usual (TAU) at end-of-therapy**.

An inspection of the funnel plot (see Figure 3) gives the impression of a tendency toward higher effect sizes for studies with a smaller sample size. The regression test for funnel plot asymmetry at *end-of-therapy* was significant (*p* = 0.017). Results of a trim and fill analysis suggest that there may be four unpublished studies on the left side of the funnel plot (see Figure 3). Including these studies in a meta-analysis would reduce the mean effect size to $\bar{d}=$ 0.14 (*SE* = 0.12).

**Figure 4:**
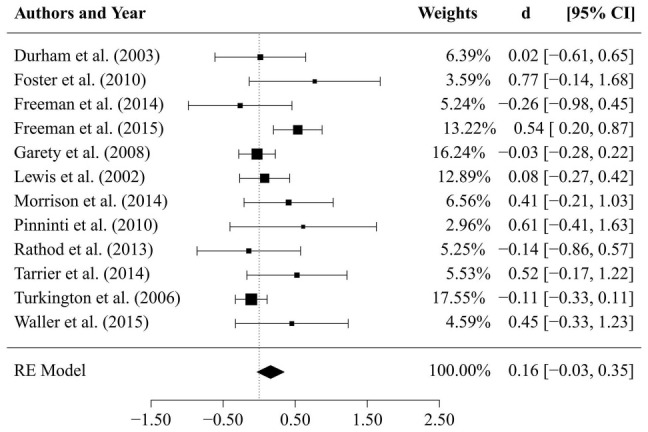
**Results of comparison between CBTp and treatment as usual (TAU) after a follow-up period of 47 weeks**.

Results of comparisons of CBTp vs. TAU (*k* = 12 studies) after an average *follow-up period* of 47 weeks are depicted in Figure 4. The estimate for the mean effect size of CBTp compared to TAU was small and non-significant ($\bar{d}\;=$ 0.16, *SE* = 0.10, *p* = 0.098, CI: −0.03, 0.35). The between-study variance was ${\hat{\tau }}^{2\;}=$ 0.04 (95%-CI: 0.00, 0.23), and the *Q*-statistic (*Q* = 18.63, *df* = 11, *p* = 0.068) was non-significant. The value of *I*^2^ = 43.38% indicated a small to medium level of heterogeneity. The regression test for funnel plot asymmetry revealed a statistically non-significant result (*p* = 0.15), thus, there was no indication of a bias. Finally, we tested whether the results of both comparisons would change if we excluded two studies that assessed specific subpopulations: patients who did not use medication (Morrison et al., 2014) and suicidal patients (Tarrier et al., 2014). However, exclusion of these studies revealed comparable mean effect sizes (CBTp vs. TAU at end-of-treatment: $\bar{d}$ = 0.32; CBTp vs. TAU at follow-up: $\bar{d}$ = 0.12).

### Comparisons of CBTp and other psychological interventions

**Figure 5:**
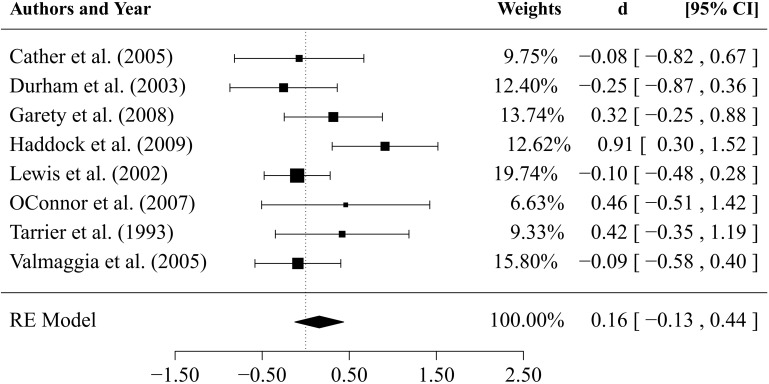
**Results of comparisons between CBTp and other psychological interventions at end-of therapy**.

The comparisons between CBTp and other psychological interventions at *end-of-therapy* (*k* = 8 studies, depicted in Figure 5) revealed an estimated mean effect size that is small and non-significant ($\bar{d}=$ 0.16, *SE* = 0.14, *p* = 0.28: 95%-CI:–0.13, 0.44). The estimator of the between-study variance was ${\hat{\tau }}^{2}=$ 0.07 (95%-CI: 0.00, 0.54). The *Q*-statistic was non-significant (*Q* = 11.69, *df* = 7, *p* = 0.111). The value of *I*^2^ = 42.1% indicated a small to medium degree of heterogeneity.

**Figure 6:**
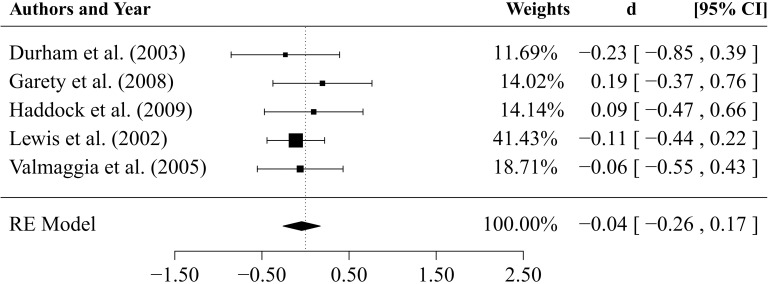
**Results of the comparison between CBTp and other psychological interventions after a follow-up period of 35 weeks**.

Results of comparisons of CBTp vs. psychological interventions (*k* = 5) after an average *follow-up* period of 34.3 weeks are depicted in Figure 6. The estimate for the mean effect size was non-significant ($\bar{d}=-0.04$, *SE* = 0.11, *p* = 0.687, 95%-CI:–0.26; 0.17). The estimated between-study variance was zero (${\hat{\tau }}^{2}=$ 0.00, 95%-CI: 0.00, 0.15) as was the *I*^2^-statistic.

### Comparison of studies that used a causal-interventionist approach and first-generation CBTp studies at end-of-therapy

In order to select newer CBTp studies, the first and the last author independently selected studies that stated in their introduction that they “used a causal-interventionist approach” or that they focused on “factors that are causally involved in the formation and maintenance of delusions.” Both consistently selected four studies, two of which focused on worrying (Foster et al., 2010; Freeman et al., 2015), one of which focused on self-esteem (Freeman et al., 2014) and one of which focused on reasoning biases (Waller et al., 2015). These studies were compared with all other studies that compared CBTp with standard treatment at *end-of-therapy* (*k* = 9: Lewis et al., 2002; Durham et al., 2003; Garety et al., 2008; Pinninti et al., 2010; Lincoln et al., 2012; Kråkvik et al., 2013; Rathod et al., 2013; Morrison et al., 2014; Tarrier et al., 2014). Results suggest a difference of 0.33 in mean effect sizes (95%-CI for the difference: −0.10, 0.75) in favor of the four studies focusing on causal factors ($\bar{d}=$ 0.51, *SE* = 0.19, *p* = 0.006), compared to all other studies ($\bar{d}=$ 0.18, *SE* = 0.11, *p* = 0.090), using an estimated between-study variance ${\hat{\tau }}^{2}=$ 0.04 within each group.

## Discussion

First, our results suggest that CBTp is more beneficial in changing delusions than standard treatment. However, the effect of CBTp on delusions did not remain stable after an average follow-up period of 47 weeks. Compared to other psychological interventions, CBTp did not prove to be better at changing delusions, neither at end-of-treatment, nor after a follow-up period. However, more recent studies that focused on factors that are hypothetically involved in the formation and maintenance of delusions rather than on the delusions *per se*, produced a numerically larger effect size of moderate magnitude compared to first-generation CBTp studies.

With regard to comparisons between CBTp and standard treatment at end-of-therapy, our results are consistent with the large body of meta-analytic research which finds small to medium effect sizes for positive symptoms (Lincoln et al., 2008; Wykes et al., 2008; Sarin et al., 2011; Jauhar et al., 2014). Moreover, our results are comparable with the recent meta-analysis by van der Gaag et al. (2014) that focused on change in delusions in individually-tailored formulation-based CBTp. However, they reported a slightly higher estimated effect size (*k* = 9; $\bar{d}$ = 0.36, 95%-CI: 0.08, 0.63) which seems to be the result of using a smaller pool of studies. The broader selection of studies in our meta-analysis produced a slightly smaller effect size; this effect size had a smaller confidence interval ($\bar{d}$ = 0.27, 95%-CI: 0.08, 0.47). Thus, the broader inclusion criteria we used lead to a slightly smaller, but also to a more precise estimation of the mean effect size of change in delusions at *end-of-therapy*. Nevertheless, we also investigated the stability of the effects, but CBTp was not more effective than standard treatment over an average follow-up period of 47 weeks. Due to the small number of RCTs that addressed both the question of change in delusions and the stability of CBTp over a follow-up period, more studies are needed to be able to draw more definite conclusions in regard to long-term effects.

It is important to note that we found a small to medium amount of variance that is due to the heterogeneity between the studies (about 42%). This variance is largely due to the study by Kråkvik et al. (2013). This study included patients with both auditory hallucinations and delusions and produced a quite large effect size ($\bar{d}$ = 0.94), which might have been influenced by the lack of blinding.

Our results seem to suggest a slightly higher effect size in smaller studies (see Figure 3). This could be due to higher motivation, engagement and team-work of therapists, more intense training, more available supervision, and fewer communication problems between researchers in smaller studies. However, a publication/reporting bias could not be ruled out. Indeed, it seems unlikely that only 19 RCTs (included in our meta-analyses) from the pool of 50 RCTs on CBTp assessed change in delusions as a secondary outcome. When having to select findings from a complex study for a publication with limited space, statistically non-significant results will probably not have the highest priority. However, in general it is difficult to distinguish bias from genuine heterogeneity in meta-analyses (Ioannidis, 2005).

It is also important to take into account that the analyses are based on mostly secondary outcome measures and effect size estimates are based on small samples resulting in low statistical power for most analyses. Further methodically rigorous studies are necessary to achieve reliable effect-size estimates.

As in the former meta-analysis by van der Gaag et al. (2014), we did not find a significant effect of CBTp compared to other psychological interventions. Consequently, we found no evidence of an advantage of CBTp compared to other interventions after an average follow-up period of 35 weeks. This could be interpreted as meaning that CBTp is not superior to other therapies such as supportive therapy, social activity therapy, problem solving or family interventions. Another explanation is that the general effect of CBTp on delusions is relatively small, making it difficult to detect an advantage of CBTp over other effective treatments, especially ones that also involve cognitive behavioral elements, such as family interventions, problem solving or social activity therapy. We may possibly have detected a slightly larger effect size if we had analyzed a larger number of studies that compared CBTp solely with placebo therapies such as supportive therapy/counseling (Lewis et al., 2002; Durham et al., 2003; Valmaggia et al., 2005) or attention placebo control (O'Connor et al., 2007). However, this was not possible given the small number of studies.

As stated above, our preliminary findings suggest a trend toward a small advantage of recent RCTs that tested a causal-interventionist approach. These studies targeted delusions specifically by focusing on factors that are hypothetically involved in the formation and maintenance of delusions compared to the first-generation CBTp approach that focuses on delusions in a more direct manner. This comparison is based on a small number of studies and the effect difference in favor of the causal-interventionist approach should be interpreted with caution. However, it is interesting to note that the causal-interventionist studies were also much shorter (requiring an average number of five sessions) than the first-generation CBTp studies, that required an average of 25 sessions. It is possible, on the one hand, that the focus on causal factors of delusions might be more beneficial than working on the delusions *per se*. On the other hand, it is also likely that shorter and more focused interventions have a positive effect because both the therapist and the patient have only a short amount of time to achieve an improvement and are thus particularly motivated and focused on the aims of the therapy. Nevertheless, it is important to note that these interventions focused specifically on delusions, whereas the first-generation CBTp studies took a broader approach, which explains why their effect sizes for the broader outcome measures such as positive symptoms or psychopathology in general tend to be numerically higher (Turner et al., 2013) than those we found for delusions in this analysis. Again, more methodologically rigorous RCTs are needed that can help us to answer these questions.

Strengths of the present study are the broader inclusion criteria resulting in inclusion of more studies and smaller confidence intervals, the focus of the study on sustainability of CBTp over a follow-up period, and the use of several statistical techniques to assess the possible influence of publication bias. Limitations are the still small number of studies that reported results on change in delusions (19 studies compared to 50 RCTs assessing the effectiveness of CBTp in schizophrenia) and the small number of studies assessing effectiveness of CBTp compared to other psychological interventions (eight studies) that resulted in low statistical power (Hedges and Pigott, 2001). In addition, one has to be aware that some comparisons in the primary studies differed in the mean number of sessions that the CBTp group received compared to the control group. However, on average, patients were offered more sessions in the comparison interventions and treatment intensity did not affect the considered outcome measures, as clarified by an explorative meta-regression.

With respect to the small number of available RCTs addressing delusions, one therefore has to be aware that the estimated mean effect size might change in a future meta-analysis after the inclusion of a small number of new studies. Moreover, it is still unknown whether patients with severe delusions are not able to participate in CBTp, as suggested by a more severe drop-out rate among them (Lincoln et al., 2012). In future studies it would be interesting to compare drop-out samples with patients who completed therapy and to ask patients who refused the treatment for their personal reasons.

To sum up, our results suggest that CBTp is superior to TAU in regard to changing delusions, but CBTp effects might not be maintained over the course of the follow-up period. Moreover, at present, CBTp is not superior to other effective interventions, neither at end-of-therapy nor after a follow-up period. Finally, interventions that focus specifically on cognitive and emotional factors that are hypothetically involved in the formation and maintenance of delusions seem to be slightly more effective and thus are a promising approach to improving interventions for delusions.

### Conflict of interest statement

The authors declare that the research was conducted in the absence of any commercial or financial relationships that could be construed as a potential conflict of interest.
