# Corrigendum: Does Cognitive Behavior Therapy for psychosis (CBTp) show a sustainable effect on delusions? A meta-analysis

**DOI:** 10.3389/fpsyg.2019.01868

**Published:** 2019-08-28

**Authors:** Stephanie Mehl, Dirk Werner, Tania M. Lincoln

**Affiliations:** ^1^Department of Psychiatry and Psychotherapy, Philipps-University Marburg, Marburg, Germany; ^2^Department of Health and Social Work, Frankfurt University of Applied Science, Frankfurt, Germany; ^3^Department of Psychological Methods and Statistics, University of Hamburg, Hamburg, Germany; ^4^Department of Clinical Psychology and Psychotherapy, University of Hamburg, Hamburg, Germany

**Keywords:** CBT, CBTp, delusions, paranoia, follow-up

In the original article, there were two errors. First, the effect size of one study (Turkington et al., 2006) was incorrect and this error resulted in an incorrect mean effect size for the comparison between Cognitive Behavior Therapy for psychosis (CBTp) and Treatment as Usual (TAU) at follow up. Further, there were errors in the classification of several studies as blind versus non-blind. These errors result in several corrections that are described, as follows.

First, the reported results and the discussion of the results in the **Abstract** section are incorrect. A correction has been made to the **Abstract** in the description of the results and the discussion.

“Cognitive Behavior Therapy for psychosis (CBTp) is an effective treatment resulting in small to medium effect sizes with regard to changes in positive symptoms and psychopathology. As a consequence, CBTp is recommended by national guidelines for all patients with schizophrenia. However, although CBTp was originally developed as a means to improve delusions, meta-analyses have generally integrated effects for positive symptoms rather than for delusions. Thus, it is still an open question whether CBTp is more effective with regard to change in delusions compared to treatment as usual (TAU) and to other interventions, and whether this effect remains stable over a follow-up period. Moreover, it would be interesting to explore whether newer studies that focus on specific factors involved in the formation and maintenance of delusions (causal-interventionist approach) are more effective than the first generation of CBTp studies. A systematic search of the trial literature identified 19 RCTs that compared CBTp with TAU and/or other interventions and reported delusions as an outcome measure. Meta-analytic integration resulted in a significant small to medium effect size for CBTp in comparison to TAU at end-of-therapy (*k* = 13; $\bar{d}$ = 0.27). However, the comparison between CBTp and TAU after an average follow-up period of 47 weeks was not statistically significant (*k* = 12, $\bar{d}$ = 0.16). When compared with other interventions, there was no significant effect of CBTp at end-of-therapy (*k* = 8; $\bar{d}$ = 0.16) and after a follow-up period (*k* = 5; $\bar{d}$ = −0.04). Comparison between newer studies taking a causal-interventionist approach (*k* = 4) and first-generation studies showed a difference of 0.33 in mean effect sizes in favor of newer studies at end-of-therapy. The findings suggest that CBTp is superior to TAU post-therapy in bringing about a change in delusions, but that this change may not be maintained over the follow-up period. Moreover, interventions that focus on causal factors of delusions seem to be a promising approach to improving interventions for delusions.”

Furthermore, the description of the included studies in the **Results** section was incorrect. Thus, a correction has been made the **Results** section, subsection **Descriptive Information on Included Studies**, paragraph two:

“Most studies (*n* = 18) used observer-rated assessments of delusions such as the Psychotic Symptom Rating Scale (*k* = 17; PSYRATS: Haddock et al., 1999a) or the Maudsley Assessment of Delusions Scale (*k* = 1; MADS: Wessely et al., 1993). Four of these studies did not use single-blind assessment (Tarrier et al., 1993; Foster et al., 2010; Kråkvik et al., 2013; Waller et al., 2015) and one study (Lincoln et al., 2012) used a self-report measure (Peters et al. Delusions Inventory: Peters et al., 1999). Most studies (*k* = 12) selectively included patients with delusions (Tarrier et al., 1993; Lewis et al., 2002; Durham et al., 2003; Valmaggia et al., 2005; O'Connor et al., 2007; Haddock et al., 2009; Foster et al., 2010; Kråkvik et al., 2013; Freeman et al., 2014, 2015; Morrison et al., 2014; Waller et al., 2015), but only one of these studies predefined change in delusions as the primary outcome (Waller et al., 2015).”

In addition, the mean effect size of the comparison between CBTp and TAU (and the corresponding statistics) after a follow-up period in the **Results** section is incorrect. Further, the reports on exclusion of studies with patients who did not use medication or suicidal patients, is incorrect. A correction has therefore been made to the **Results** section, subsection **Comparison of CBTp and Treatment as usual (TAU)**, paragraph three:

“Results of comparisons of CBTp vs. TAU (*k* = 12 studies) after an average *follow-up period* of 47 weeks are depicted in Figure 4. The estimate for the mean effect size of CBTp compared to TAU was small and non-significant ($\bar{d}\;=$ 0.16, *SE* = 0.10, *p* = 0.098, CI: −0.03, 0.35). The between-study variance was ${\hat{\tau }}^{2\;}=$ 0.04 (95%-CI: 0.00, 0.23), and the *Q*-statistic (*Q* = 18.63, *df* = 11, *p* = 0.068) was non-significant. The value of *I*^2^ = 43.38% indicated a small to medium level of heterogeneity. The regression test for funnel plot asymmetry revealed a statistically non-significant result (*p* = 0.15), thus, there was no indication of a bias. Finally, we tested whether the results of both comparisons would change if we excluded two studies that assessed specific subpopulations: patients who did not use medication (Morrison et al., 2014) and suicidal patients (Tarrier et al., 2014). However, exclusion of these studies revealed comparable mean effect sizes (CBTp vs. TAU at end-of-treatment: $\bar{d}$ = 0.32; CBTp vs. TAU at follow-up: $\bar{d}$ = 0.12).”

**FIGURE 4:**
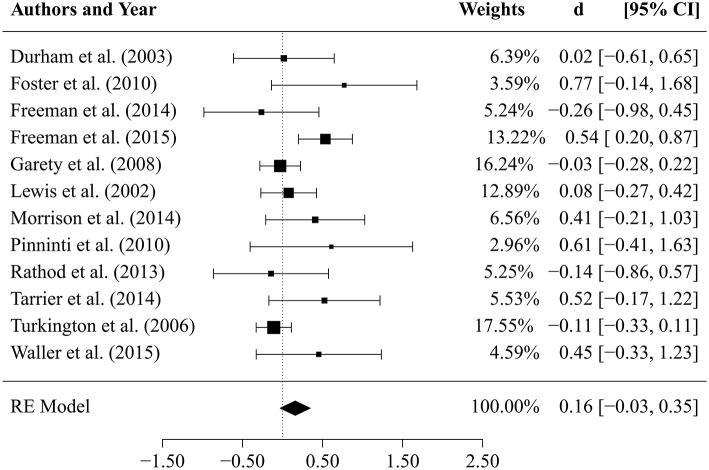
Results of comparison between CBTp and treatment as usual (TAU) after a follow-up period of 47 weeks.

Further, the results of the comparison between CBTp and TAU after a follow-up period were presented incorrectly in the **Discussion** section. A correction has therefore been made to the **Discussion** section, paragraph one:

“First, our results suggest that CBTp is more beneficial in changing delusions than standard treatment. However, the effect of CBTp on delusions did not remain stable after an average follow-up period of 47 weeks. Compared to other psychological interventions, CBTp did not prove to be better at changing delusions, neither at end-of-treatment, nor after a follow-up period. However, more recent studies that focused on factors that are hypothetically involved in the formation and maintenance of delusions rather than on the delusions *per se*, produced a numerically larger effect size of moderate magnitude compared to first-generation CBTp studies.”

An additional correction has been made to the **Discussion** section, paragraph two:

“With regard to comparisons between CBTp and standard treatment at end-of-therapy, our results are consistent with the large body of meta-analytic research which finds small to medium effect sizes for positive symptoms (Lincoln et al., 2008; Wykes et al., 2008; Sarin et al., 2011; Jauhar et al., 2014). Moreover, our results are comparable with the recent meta-analysis by van der Gaag et al. (2014) that focused on change in delusions in individually-tailored formulation-based CBTp. However, they reported a slightly higher estimated effect size (*k* = 9; $\bar{d}$ = 0.36, 95%-CI: 0.08, 0.63) which seems to be the result of using a smaller pool of studies. The broader selection of studies in our meta-analysis produced a slightly smaller effect size; this effect size had a smaller confidence interval ($\bar{d}$ = 0.27, 95%-CI: 0.08, 0.47). Thus, the broader inclusion criteria we used lead to a slightly smaller, but also to a more precise estimation of the mean effect size of change in delusions at *end-of-therapy*. Nevertheless, we also investigated the stability of the effects, but CBTp was not more effective than standard treatment over an average follow-up period of 47 weeks. Due to the small number of RCTs that addressed both the question of change in delusions and the stability of CBTp over a follow-up period, more studies are needed to be able to draw more definite conclusions in regard to long-term effects.”

A correction has also been made to the Discussion section, paragraph three:

“It is important to note that we found a small to medium amount of variance that is due to the heterogeneity between the studies (about 42%). This variance is largely due to the study by Kråkvik et al. (2013). This study included patients with both auditory hallucinations and delusions and produced a quite large effect size ($\bar{d}=0.94$), which might have been influenced by the lack of blinding.”

A correction has also been made to the **Discussion** section, paragraph ten:

“To sum up, our results suggest that CBTp is superior to TAU in regard to changing delusions, but CBTp effects might not be maintained over the course of the follow-up period. Moreover, at present, CBTp is not superior to other effective interventions, neither at end-of-therapy nor after a follow-up period. Finally, interventions that focus specifically on cognitive and emotional factors that are hypothetically involved in the formation and maintenance of delusions seem to be slightly more effective and thus are a promising approach to improving interventions for delusions.”

Furthermore, the effect size of the Turkington study (Turkington et al., 2006) was incorrect. The correct effect size is: $\bar{d}$ = −0.11 (−0.33, 0.11). Furthermore, the mean effect size of the last row of the RE Model is incorrect. The correct effect size is: $\bar{d}$ = 0.16 (−0.03, 0.35).

Thus, Figure 4 has been corrected.

Furthermore, in Table 1, four studies (Tarrier et al., 1993; Lincoln et al., 2012; Kråkvik et al., 2013 and Waller et al., 2015) were incorrectly described as studies with blind assessment and their blinding status should be described as non-blinded (“No”). Further, one study (‘O’Connnor) was incorrectly described as non-blind and its blinding status should be described as blinded (“Yes”).

**Table 1:** Studies included in the comparison of CBTp vs. TAU and CBTp vs. other psychological interventions: description of the intervention, patient characteristics and outcome measure.

**Author and Year**	**Subject characteristics: Experimental Condition (EC), Control Condition I (C1) Control Condition II (CCII)**	**Experimental condition (EC) CBT format patients**	**Control condition I (CC I) format patients**	**Control condition II (CC II)**	**Duration of intervention EC/CCI/CC II**	**Total no. of sessions, Mean number of sessions, EC/CC I/CC II**	**Selected outcome measure**	**Blind assessment?**	**ITT-data?**	**Follow-up**
Cather et al., 2005	Number of randomized patients: *n* = 28, Diagnoses: 17 SZ; 11 SA, Age: EC: *M* = 45.8 (*SD* = 10.2) CCI: *M* = 33.1 (*SD* = 10.3), Medication: EC: 100%/CCI: 100%	Functional CBT, Based on established manuals (Kingdon and Turkington, 1994; Fowler et al., 1995; Chadwick et al., 1996; Nelson, 1997), Number of randomized patients: *n* = 15	Psychoeducation, Number of randomized patients: (*n* = 15)		16/16 weeks	Total number of sessions: 16/16^6^	PSYRATS del.	Yes	No	–
Durham et al., 2003	Number of randomized patients *n* = 66, Diagnoses: 59 SZ; 5 SA; 2 DD, Age: EC: *M* = 36 (*SD* = 10.0)/CCI: *M* = 36 (*SD* = 10.2)/CC II: *M* = 37 (*SD* = 11.2), Medication: EC: 100%/CC I: 86%	CBT, Best practice based on established manuals (Tarrier, 1992; Kingdon and Turkington, 1994), Number of randomized patients: *n* = 22	TAU, Number of randomized patients: *n* = 21	Supportive therapy, Number of randomized patients: *n* = 23	39 weeks/–/22 weeks	Total number of sessions: EC: 20/–/CC II: 20, Mean number of sessions: EC: 14.8,/–/CC II: 16.8, D_sessions_ = −2.0	PSYRATS del.	Yes	No	52 weeks
Foster et al., 2010	Number of randomized patients *n* = 24, Diagnoses: SZ, SA, and DD^1^, Age: EC: 40.0 (10.5)/CC I: 39.1 (9.2), Medication: EC: 92%/CCI: 83%	Worry-CBT, Fixed sessions based on a manual (Wells, 1997), Number of randomized patients: *n* = 12	TAU, Number of randomized patients: *n* = 12		4 weeks/–	Total number of sessions: 4/–	PSYRATS del.	No	No	9 weeks
Freeman et al., 2015	Number of randomized patients: *n* = 150, Diagnoses: 111 SZ; 11 SA; 10 DD; 18 POS, Age: EC: 40.9 (10.5)/CC I: 42.1 (13.1), Medication: 94%^3^	Worry-CBT, Based on self-help manual, (Freeman and Freeman, 2013), Number of randomized patients: *n* = 73	TAU, Number of randomized patients: *n* = 77	–	8 weeks./–	Total number of sessions: 6/–, Mean number of sessions: EC: 5.5	PSYRATS del.	Yes	No	24 weeks
Freeman et al., 2014	Number of randomized patients: *n* = 30, Diagnoses: 22 SZ; 6 SA; 1 DD; 1 POS, Age: EC: 41.9 (11.5)/CC I: 41.5 (13.1), Medication: EC: 100%/CC I: 100%	Brief CBT, Based on self-help manual (Freeman and Freeman, 2012), Number of randomized patients: *n* = 15	TAU, Number of randomized patients: *n* = 15		8 weeks/–	Total number of sessions: 6/–, Mean number of session: EC: 6.67/–	PSYRATS del.	Yes	No	12 weeks
Garety et al., 2008	Number of randomized patients: *n* = 328, Diagnoses: 258 SZ; 38 SA; 5 DD, Age: n.r., Medication: n.r.	CBT (carer + no-carer), Based on an established manual (Fowler et al., 1995), Number of randomized patients: *n* = 160	TAU, Number of randomized patients (carer + no-carer): *n* = 140	Family intervention, Number of randomized patients: *n* = 28	39 weeks	Total number of sessions: 20/–, Mean number of sessions: EC: 14.3/–/CC II: 13.9, D_sessions_ = 0.4	PSYRATS del., conviction and delusion distress	Yes	No	52 weeks
Haddock et al., 2009	Number of randomized patients: *n* = 77, Diagnoses: 69 SZ; 7 SA; 1 POS, Age: EC: 35.7 (12.5)/CC I: 33.9 (9.7), Medication: EC: 100%/CC I: 100%	CBT, Based on an established manual (Haddock et al., 2004), Number of randomized patients: *n* = 38	Social activity therapy, Number of randomized patients: *n* = 38		26 weeks	Total number of sessions: 25, Mean number of sessions: EC: 13.13/CC I: 14.9, D_sessions_ = −1.77	PSYRATS del.	Yes	No	24 weeks
Kråkvik et al., 2013	Number of randomized patients: *n* = 55, Diagnoses: 34 SZ/2 SA/9 DD, Age: EC: 37.5 (11.2)/ CC I: 35.3 (8.9), Medication: EC: 100%/CC I: 100%	CBT, Simplified version of an established manual (Chadwick et al., 1996), Number of randomized patients: *n* = 23	TAU^2^, Number of randomized patients: *n* = 22	–	26 weeks	Total number of sessions: 20	PSYRATS cognitive and emotional	No	Yes	52 weeks^2^
Lewis et al., 2002	Number of randomized patients: *n* = 309, Diagnoses: 123 SZ; 109 SFD; 39 SA; 25 DD; 13 POS, Age: EC: 29.1/CC I: 27.0/CC II: 27.2^4^, Medication: EC: 100%/CC I: 100%/CC II: 100%	CBT, Based on an established manual (Haddock et al., 1999b), Number of randomized patients: *n* = 101	TAU, Number of randomized patients: *n* = 102	Supportive counseling Number of randomized patients: *n* = 106	5 weeks	Total number of sessions: 20, Mean number of sessions:EC: 16.1/–/CC II: 15.7, D_sessions_ = −0.4	PSYRATS del.	Yes	No	67 weeks
Lincoln et al., 2012	Number of randomized patients: *n* = 80, Diagnoses: 58 SZ; 13 SA; 5 DD; 4 APD, Age: EC: 33.2 (10.4)/CC I: 33.1 (10.9), Medication: EC: 100%/CC I: 97%	CBTp, Based on an established German manual (Lincoln, 2006), Number of randomized patients: *n* = 40	TAU^2^, Number of randomized patients: *n* = 40	–	38 weeks	No fixed number of sessions. Mean number of sessions EC: 29/–	PDI distress, preoccupation, conviction	No	Yes	52 weeks^2^
Morrison et al., 2014	Number of randomized patients: *n* = 74, Diagnoses: SZ, SA, and DD^1^, Age: EC: 33.0 (13.1)/CC I: 29.7 (11.9), Medication: EC: 0%/CC I: 0%	CBTp, Based on established manuals (Morrison et al., 2004; Kingdon and Turkington, 2005), Number of randomized patients: *n* = 37	TAU, Number of randomized patients: *n* = 37	–	39 weeks	Total number of sessions: 26, Mean number of sessions: EC: 13.3/–	PSYRATS cognitive and emotional	Yes	No	19 weeks
O'Connor et al., 2007	Number of randomized patients: *n* = 24, Diagnoses: 24 DD, Age: EC: 40.0 (9.4)/CC I: 36.8 (13.5), Medication: EC: 100%/CC I: 100%	CBTp, Based on established manuals (Fowler et al., 1995; Chadwick et al., 1996), Number of randomized patients: *n* = 12	Attention placebo control, Number of randomized patients: *n* = 12	–	24 weeks	Total number of sessions: 24	MADS	Yes	No	–
Pinninti et al., 2010	Number of randomized patients: *n* = 33, Diagnoses: 11 SZ; 22 SA, Age: 40.0 (11.0)^3^, Medication: EC: 100%/CC I: 100%	CBTp, Not manualized, Number of randomized patients: *n* = 18	TAU, Number of randomized patients: *n* = 15	–	12 weeks	Total number of sessions: 12, Mean number of sessions EC: 11.9/–	PSYRATS del.	Yes	No	24 weeks
Rathod et al., 2013	Number of randomized patients *n* = 35, Diagnoses: SZ, SA, and DD^1^, Age: EC: 31.4 (12.3)/CC I: 35.6 (10.7), Medication: EC: 100%/CC I: 100%	Culturally adapted CBTp Based on a study protocol (Rathod et al., 2010), Number of randomized patients: *n* = 17	TAU, Number of randomized patients: *n* = 15	–	18 weeks	Total number of sessions: 16, Mean number of sessions: EC: 13.6/–	CPRS del.	Yes	Yes	26 weeks
Tarrier et al., 1993	Number of randomized patients: *n* = 27, Diagnoses: 307 SZ, Age: EC: 42.8 (12.3)/CC I: 42.8 (12.3), Medication: EC: 100%/CC I: 100%	Coping strategy enhancement, Based on an established manual (Tarrier, 1992), Number of randomized patients: *n* = 15	Problem solving, Number of randomized patients: *n* = 12	–	5 weeks	Total number of sessions: 10	PAS delusions	No	No	31 weeks
Tarrier et al., 2014	Number of randomized patients *n* = 49, Diagnoses: SZ, SA, DD, POS^1^, Age: EC: 32.6 (11.7)/CC I: 37.3 (14.2), Medication: EC: 100%/CC I: 100%	CBT for suicidal patients, Based on a manual (Tarrier et al., 2013), Number of randomized patients: *n* = 25	TAU, Number of randomized patients: *n* = 24	–	12 weeks	Total number of sessions: 24	PSYRATS del.	Yes	No	17 weeks
Turkington et al., 2006	Number of randomized patients: *n* = 422, Diagnoses: 422 SZ, Age: n. r., Medication: EC: 100%/CC I: 100%	CBTp, Based on established manuals (Kingdon and Turkington, 1994, 2005), Number of randomized patients: *n* = 281	TAU^2^, Number of randomized patients: *n* = 141	–	10.5 weeks	Total number of sessions: Mean number of sessions: EC: 6/–	PSYRATS del.	Yes	No	52 weeks
Valmaggia et al., 2005	Number of randomized patients: *n* = 62, Diagnoses: 62 SZ, Age: EC: 35.4 (10.5)/CC I: 35.5 (11.4), Medication: EC: 100%/CC I: 100%	CBTp, Based on an established manual (Kingdon and Turkington, 1994), Number of randomized patients: *n* = 36	Supportive counseling, Number of randomized patients: *n* = 26	–	22 weeks	Total number of sessions: 16	PSYRATS cognitive and emotional scale	Yes	Yes	48 weeks
Waller et al., 2015	Number of randomized patients: *n* = 31, Diagnoses: 27 SZ, 2 SA, 2 DD, Age: EC: 39.1 (10.5)/CC I: 43.0 (10.7), Medication: EC: 90%/CC I: 91%	Focused CBT, Sessions described in the study, Number of randomized patients: *n* = 20	TAU, Number of randomized patients: *n* = 11	–	5 weeks	Total number of sessions: 4	PSYRATS del.	No	Yes	8 weeks

M, Mean; SD, Standard deviation; TAU, Treatment as Usual; EC, Experimental condition; CCI, Control condition I; CCII, Control condition II; SZ, Schizophrenia; SA, Schizoaffective Disorder; DD, Delusional disorder; APD, Acute psychotic disorder; POS, Psychosis not otherwise specified; SFD, Schizophreniform disorder;Medication, percentage of patients treated with antipsychotic medication. PSYRATSdel., PSYRATS delusions score; CPRS, Comprehensive Psychopathology Rating Scale; PAS, Psychiatric Assessment Scale;n.r., not reported;Dsessions, Mean number of CBT sessions—Mean number of other therapy sessions;

1 no information on diagnosis ratio;

2 study was not included in follow-up comparison between CBTp and TAU,as the study used a wait-list design and comparisons between CBTp and TAU are not possible at follow-up assessment;

3 variable was only reported for all patients;

4 *SD was not reported*.

Thus Table 1 has been corrected.

The authors apologize for these errors and would like to thank Prof. Dr. Laws for pointing them out (Laws, 2016). Unfortunately, we did not receive notice of the comment by Prof. Dr. Laws until this year (2019). In response, we have corrected the errors, revised the discussion of the article and changed the scientific conclusions that were influenced by these errors. The original article has been updated.
